# Comparing the impact of sample multiplexing approaches for single-cell RNA-sequencing on downstream analysis using cerebellar organoids

**DOI:** 10.1016/j.isci.2026.114780

**Published:** 2026-01-22

**Authors:** Kseniia Sarieva, Theresa Kagermeier, Vladislav Lysenkov, Francesco Castagnetti, Zeynep Yentuer, Katharina Becker, Julia Matilainen, Nicolas Casadei, Simone Mayer

**Affiliations:** 1 Hertie Institute for Clinical Brain Research, University of Tübingen, 72076 Tübingen, Germany; 2 International Max Planck Research School, Graduate Training Centre of Neuroscience, University of Tübingen, 72076 Tübingen, Germany; 3 Institute of Medical Genetics and Applied Genomics, University Hospital Tübingen, 72076 Tübingen, Germany; 4 NGS Competence Center Tübingen, 72076 Tübingen, Germany; 5 Heidelberger Akademie der Wissenschaften, 69117 Heidelberg, Germany; 6 IMPRS, The Mechanisms of Mental Function and Dysfunction, University of Tübingen, 72076 Tübingen, Germany; 7 German PCH Patient Network (PCH-Familie e.V.), 71034 Böblingen, Germany; 8 Zoological Institute, Karlsruhe Institute of Technology (KIT), 76131 Karlsruhe, Germany; 9 Institute of Biological and Chemical Systems - Functional Molecular Systems, Karlsruhe Institute of Technology (KIT), 76131 Karlsruhe, Germany

**Keywords:** natural sciences, biological sciences, cellular neuroscience, cell biology

## Abstract

Multiplexing overcomes limited throughput in single-cell RNA sequencing (scRNA-seq). Commercial strategies include Parse Biosciences combinatorial barcoding (Parse) and 10x Genomics CellPlex with microfluidic capture (10x). It is currently unknown how these techniques differ when characterizing complex tissues. Cerebellar organoids are a highly relevant model for studying cerebellar evolution, development, and disease. Yet, their extensive characterization through scRNA-seq is ongoing. Therefore, we compared the two multiplexing techniques using cerebellar organoids. While both strategies demonstrated technical reproducibility and revealed comparable cellular diversity, we found more stressed cells in 10x than in Parse. Additionally, Parse covered a higher gene biotype diversity and showed lower mitochondrial and ribosomal protein-coding transcript fractions. In summary, we demonstrate that both techniques provide similar insight into cerebellar organoid biology, but the flexibility of experimental design, capture of long transcripts, and the level of cell stress caused by the two workflows differ.

## Highlights

• 10x yields more genes than Parse and more ribosomal and mitochondrial gene reads
• The Parse workflow results in lower cell stress than 10x
• Data from both technologies can be successfully integrated
• 10x and Parse capture the same cellular diversity in human cerebellar organoids

## Graphical abstract

**Figure:**
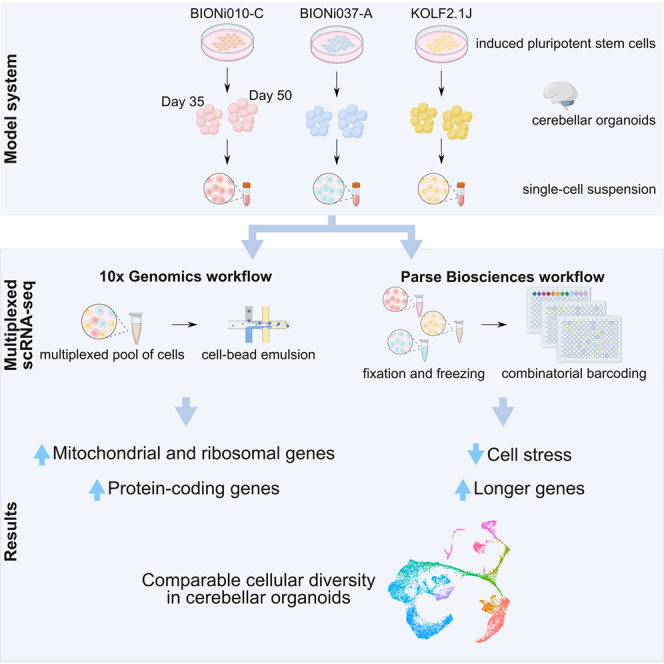

## Teaser

Natural sciences; Biological sciences; Cellular neuroscience; Cell biology; Omics; scRNA-seq; brain region-specific neural organoids; cerebeller organoids

## Introduction

Single-cell RNA-sequencing (scRNA-seq) has revolutionized our approach to characterize cell types, states, and lineages in various biological systems and is increasingly used in drug screening. While biological replica is essential for robust statistical analysis and the detection of even subtle changes between experimental conditions, replication has often been limited by technically challenging workflows and high costs.^1^^,^^2^ Additionally, effective cell sampling maximizes the capture of cellular heterogeneity including rare cell populations.^3^ Recent advances in commercialized kits now allow sample multiplexing, increasing both the number of cells assayed and the number of possible biological replicates. While combinatorial barcoding (as provided commercially by Parse Biosciences, hereafter Parse) is inherently multiplexed, microfluidic approaches (as provided commercially by 10x Genomics, hereafter 10x) require an additional labeling step for barcoding, mediated by antibodies or lipids.^4^ However, increasing the number of samples remains technically challenging when working with fresh tissue because dissociation, a highly manual process, needs to be parallelized.^5^ Fixation of the dissociated cells before capture (as performed in the Parse workflow) overcomes this obstacle, and different samples, for instance from different experimental time points, can be sequenced together, thereby avoiding batch effects of the capture. The kits allow multiplexing of up to 12 (10x) or 96 samples (Parse). The higher the number of multiplexed samples, the lower are the per-sample costs of cell capture with both strategies.

Since scRNA-seq multiplexing is widely used and datasets from different studies and experimental approaches are increasingly compared and integrated, it is important to consider the effects of the chosen multiplexing approach on the results. A recent study comparing both technologies using peripheral blood mononuclear cells (PBMCs) demonstrated that Parse had a higher sensitivity for detecting rare cell types.^6^ Furthermore, it was shown that Parse covered a wider range of gene lengths, and that 10x was biased toward more granule cell (GC)-rich transcripts.^6^ However, it remains unclear, to what extent these differences affect downstream analysis and highly complex 3D samples that require dissociation such as neural organoids.

Regionalized neural organoids recapitulate the development of specific brain regions with their specialized neural cell populations, making them a particularly powerful tool to study human neurodevelopment,^7^ to model neurological disorders,^8^^,^^9^ and to test on- and off-target effects of pharmaceuticals.^10^^,^^11^ The human cerebellum has long been thought to mainly be involved in motor learning and coordination,^12^ however, more recent insights into cerebellar function, describe its major contribution to cognitive functions such as attention, task execution, working memory, language and social behavior,^13^ and a contribution to neurodevelopmental disorders (NDD) such as autism spectrum disorder (ASD).^14^^,^^15^ Two cerebellar progenitor zones, the ventricular zone (VZ) and the rhombic lip (RL), arise from the rhombencephalon.^16^^,^^17^ The VZ gives rise to all inhibitory neurons of the future cerebellum, including Purkinje cells (PCs) and inhibitory neurons of the deep cerebellar nuclei. The RL generates all excitatory neurons, including GCs and excitatory neurons of the deep cerebellar nuclei.^18^ Progenitors and neurons from both progenitor zones can now be generated in human cerebellar organoids, placing them in a unique position to model cerebellar disorders such as cerebellar hypoplasias, Dandy-Walker Syndrome, ataxias, and medulloblastoma as pioneered in several recent studies.^9^^,^^19^^,^^20^ However, the protocols underlying their generation are still being improved,^21^^,^^22^^,^^23^ and few single-cell RNA datasets of selected cell lines are available.^21^^,^^23^^,^^24^

Here, we addressed two important gaps in knowledge related to multiplexing in scRNAseq and cerebellar organoid generation by comparing the technical features between the two multiplexing strategies, Parse and 10x, in complex tissue-like samples, cerebellar organoids derived from three control iPSC lines at two time points.

## Results

### Experimental design and quality assessment

**Figure 1:**
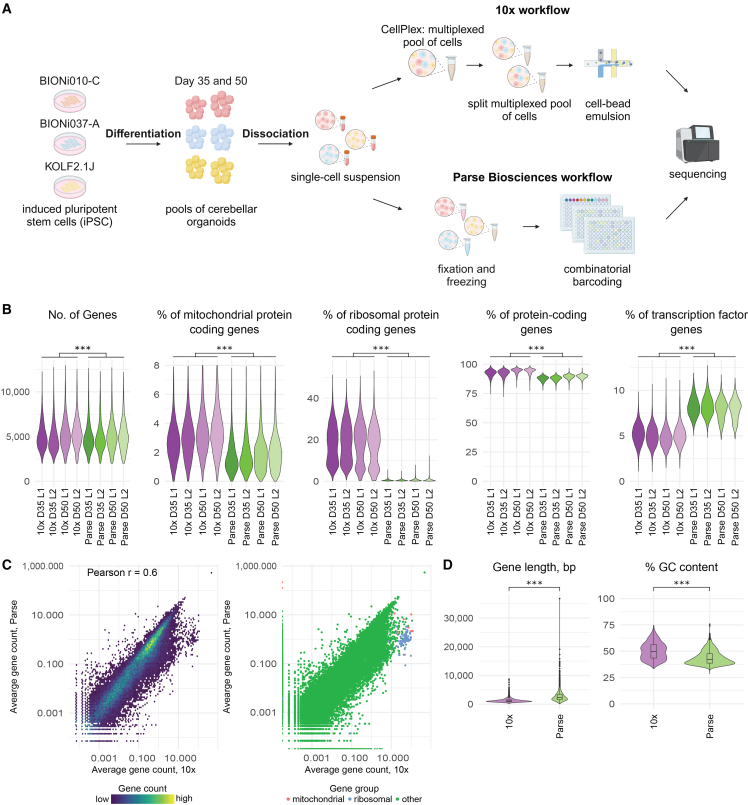
Study design, quality control, and potential biases in the data (A) Three iPSC lines (BIONi010-C, BIONi037-A, and KOLF2.1J) were differentiated to cerebellar organoids until days 35 and 50. The organoids generated from the same cell line were pooled and dissociated into single cells when each single-cell suspension was split into two portions. One set of single-cell suspensions was immediately subjected to sample multiplexing with CellPlex and processed in 10x Genomics 3′GEX+FB pipeline. The second set of single-cell suspensions was frozen until all samples were available. The samples were further processed though Parse Biosciences Evercode v2 pipeline. Libraries were sequenced, and the resulting FASTQ files were processed with technology-specific computational pipelines. Count matrices were further analyzed. Graphic was created with BioRender.com. (B) Quality statistics after quality control. Color represents sample identity with respect to technology (10x or Parse), day of differentiation (D35 or D50), and library (L1 or L2). 10x, *n* = 29,505, Parse, *n* = 14,542 cells. Three-way ANOVA, *p* values represent differences between technologies, ∗∗∗*p* < 0.001. (C) Left, density scatterplot showing correlation of average gene expression between the two technologies. Right, scatterplot showing correlation of average gene expression between the two technologies. Color represents gene group. (D) Distributions of gene GC content and gene length for differentially expressed genes between technologies. Two-sided *t* test, ∗∗∗*p* < 0.001. See also Figures S1–S3 and Tables S1 and S2.

To assess the reproducibility of cerebellar organoid differentiation and comparability of two multiplexed scRNA-seq methods, we differentiated three iPSC lines (BIONi010-C, BIONi037-A, and KOLF2.1J) into cerebellar organoids (Figure 1A). Cell lines were handled in parallel throughout the experimental period. On day 28 (D28) and day 42 (D42) of differentiation, organoids were collected for quality control assessment by immunohistochemistry. We observed the expression of Purkinje cell marker SKOR2 at both time points (Figure S1A), the GC precursor markers BARHL1 and ATOH1 were expressed at D42 (Figure S2C), and general neuroectodermal commitment was indicated by the presence of neural precursor marker SOX2 as well as the early pan-neuronal markers Tuj1 (D28 and D42; Figures S1C and S2B) and MAP2 (D42; Figure S2B). Further, all cell lines demonstrated cell division at both timepoints, indicated by the expression of Ki-67 (Figures S1B and S2A). Samples for scRNA-seq were harvested on day 35 (D35) and day 50 (D50) of differentiation. Pools of 24 organoids per cell line and time point were dissociated. One aliquot of each cell suspension was used for 10x, the other for Parse scRNA-seq workflow. This experimental design minimized the effect of biological variability and focused on technical differences between 10x and Parse.

Libraries were sequenced to achieve over 50,000 reads per cell (Table S1), and raw FASTQ files were downsampled to 50,000 reads per cell to allow a direct comparison of gene detection sensitivity (Table S1). Alignment of reads was performed through technology-specific pipelines: cellranger v7.2.0 *multi* pipeline (10x) and split-pipe v1.1.2 (Parse).

In both technologies, most reads were mapped to the genome (93.2% for 10x, 91.8% for Parse; Figure S3A and Table S2), with exonic reads constituting 56.3% of all reads in 10x, and 30.1% in Parse (Figure S3A and Table S2). Valid barcodes were identified for 97.2% of reads for 10x and 79.9% for Parse (Figure S3A and Table S2). The cell recovery rate was 42.7% for 10x and 16.5% for Parse (Figure S3B and Table S2).

For further comparisons, technology-specific cell-by-gene matrices were merged. We found that 32,408 genes had a non-zero expression in both technologies, while 2,159 and 12,098 genes were uniquely expressed in 10x and Parse, respectively (Figure S3C). After merging count matrices from both technologies, we only retained genes that had a non-zero expression in more than 8 cells, resulting in the count matrix with 38,580 genes (Figure S3D).

For further analysis, we used the following combination of metadata parameters to assign cells to samples unless stated otherwise: (1) technology (10x vs. Parse); (2) day of differentiation (D35 vs. D50) of cerebellar organoids; and (3) sequencing library (L1 and L2). Day of differentiation was used as a covariate to acknowledge both biological differences in the stage of organoid differentiation and technical differences arising from harvesting D35 and D50 samples on different days. The sequencing library was used as a covariate to show the reproducibility of the workflow within each technology.

After cell-level quality control (QC), we recovered 87.2% of cells from 10x and 95.6% of cells from Parse datasets (10x, 29,505 out of 33,951 cells; Parse, 14,542 out of 15,226 cells; Figure S3E). Interestingly, the number of genes per cell was higher in Parse both before and after QC (*p* < 0.001; Figure 1B). While protein-coding genes were the most abundant in both technologies (Figure 1B), Parse recovered a higher proportion of non-coding RNAs (ncRNA) reads, including long non-coding RNA (lncRNA) (Figure S3F). Additionally, the percentage of mitochondrial and ribosomal protein-coding transcripts was lower in Parse than in 10x. In contrast, the percentage of reads originating from transcription factors (TFs) among protein-coding genes was higher in Parse than in 10x (Figures 1B and S3E). In line with previous findings,^6^ the correlation of gene expression between the two technologies across cells was only moderate (Pearson’s *r* = 0.6) (Figure 1C), indicating differential gene detection between the two technologies.

Different RNA-seq technologies are known to have biases in gene detection based on gene properties such as GC content and gene length.^6^^,^^25^ To characterize these biases, we analyzed the correspondence between gene abundance and gene length or GC content (Figures 1D and S3F). While using all expressed genes per technology revealed small but statistically significant differences in these parameters (*p* < 0.001; Figure S3F), gene length, and GC content of differentially expressed genes (DEG) per technology (10x, 2,737 DEGs; Parse, 4,055 DEGs) differed to a higher extent (Figure 1D), reminiscent of previously published results.^6^ We observed a bias toward detecting longer genes in Parse, both for protein-coding genes and lncRNA (Figure S3G). Finally, we performed an extensive analysis of gene detection sensitivity and biases (Table S2) largely corroborating results from the previous benchmarking study.^6^ We therefore suggest that the observed differences are characteristic features of 10x and Parse technologies independent of sample type.

### Technical and biological differences between technologies

**Figure 2:**
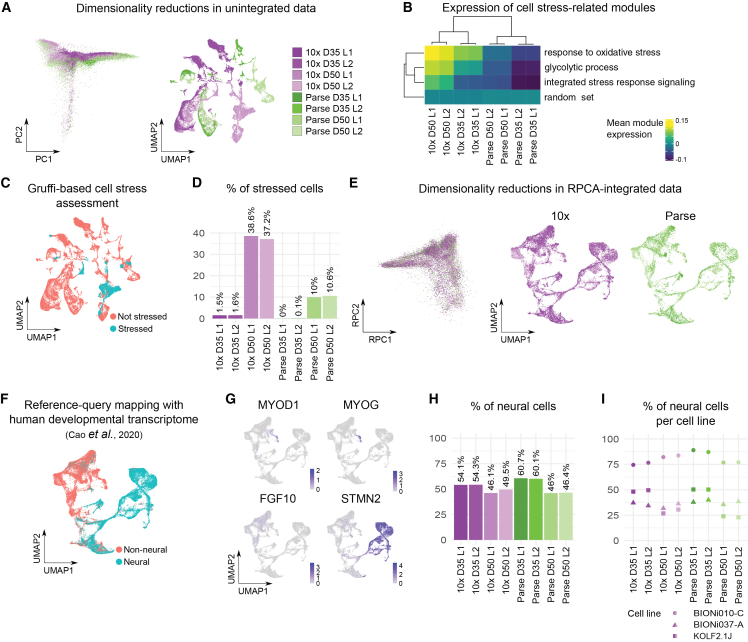
Assessment of neural lineage identity (A) PCA and UMAP plots for globally normalized and unintegrated data. (B) Heatmap representing mean module expression scores of GO terms related to aspects of cell stress. (C) UMAP plot representing cell stress status of cells based on Gruffi assessment. (D) Percentage of stressed cells based on Gruffi assessment. (E) RPCA and UMAP plots for globally normalized and RPCA-integrated data originating from non-stressed cells. (F) UMAP plot representing neural lineage status of cells based on reference-query integration with human developmental transcriptome.^26^ (G) Feature plots showing expression of selected genes to highlight developmental lineages. (H) Percentage of neuroectodermal cells based on reference-query integration with human developmental transcriptome. (I) Percentage of neuroectodermal cells per cell line based on reference-query integration with human developmental transcriptome. For (A, D, E, H, and I), color represents sample identity with respect to technology (10x or Parse), day of differentiation (D35 or D50), and library (L1 or L2). See also Figure S3 and Table S3.

Next, data normalization revealed highly variable genes for principal component analysis (PCA) as well as uniform manifold approximation and projection (UMAP) on unintegrated data (Figure 2A). As expected from previous results^6^ and our QC, both PCA and UMAP revealed major differences between the technologies (Figure 2A). We hypothesized that these differences arise from sample preparation where cells for Parse were immediately fixed and frozen after dissociation, while cells undergoing 10x capture were depleted of nutrients and passed through microfluidic channels of the instrument before lysis.

Hence, we hypothesized that cellular stress may contribute to differences between samples. We analyzed the expression of gene ontology (GO) modules involved in different modalities of cellular stress and its downstream effects, such as integrated stress response (ISR) (Figure S4A). In hierarchical clustering of average GO module expression scores, samples from the two technologies clustered apart. The major differences came from three terms: response to oxidative stress, glycolytic process, and ISR signaling (Figure S4A). Using only these three modules and the random set for hierarchical clustering led to the same results (Figures 2B and S4A).

We further determined the number of stressed cells using Gruffi^27^ using the top cell stress terms from the module expression analysis: glycolytic process (GO:0006096) and ISR signaling (GO:0140467). We found that the percentage of stressed cells varied between technologies but also between days of organoid differentiation (Figures 2C, 2D, and S4B). There were more stressed cells in the 10x data and both technologies captured more stressed cells in D50 cerebellar organoids (Figure 2D). This finding can be explained by the diffusion-based distribution of nutrients in organoids leading to an increasing nutrient deficiency as organoids grow (D50 vs. D35).^28^^,^^29^ We therefore removed cells that were classified as stressed by Gruffi (6,595 out of 44,047 cells that passed QC) from further analysis, integrated normalized counts by sample using reciprocal PCA, and repeated PCA and UMAP. This analysis revealed that the data from the two technologies can be easily integrated (Figure 2E).

To analyze the biological reproducibility of the cerebellar organoid protocol between different iPSC lines, we characterized the cellular diversity. We first aimed to understand whether organoids had neural identity. We, therefore, performed reference-query mapping of our dataset onto the human developmental transcriptome using Azimuth.^26^^,^^30^ This reference dataset includes cell types from various tissues, including the nervous system and the cerebellum. We first assigned our cells with cell types from the reference dataset featuring cells from 15 human organs between 72 and 129 days post-conception^26^^,^^30^ (Figure S4C). High prediction scores were assigned to the cells annotated as skeletal muscle, bronchiolar and alveolar epithelial cells, enteric nervous system glia, astrocytes, and some neuronal cells (Figure S4D). However, prediction scores varied between cells (0.59 ± 0.26, mean ± SD; Figure S4D), with most cells not reaching a high-confidence prediction score of 0.75.^30^ Therefore, we did not rely on the annotation of certain cell types but grouped the cells into two categories—neural and non-neural (Figures 2F and Table S3). We found a considerable portion of cells having non-neural identity (Figure 2F) with subsets of cells expressing muscular markers (e.g., *MYOD1* and *MYOG*^31^) and endo-/mesodermal markers (e.g., *FGF10*^32^) (Figure 2G). Accordingly, cells expressing muscular markers were annotated as muscular cells with high confidence (Figure S4D). In contrast, most cells classified as neural expressed the pan-neuronal marker *STMN2* (Figure 2G). Among those cells, there were cells annotated as granule neurons and Purkinje neurons, albeit with lower prediction scores (Figures S4C and S4D). Overall, the proportion of neural cells ranged from 46.0% to 60.7% per sample (Figure 2H). Importantly, considerable differences were observed between the three iPSC lines that the organoids were generated from with BIONi010-C cell line having the highest number of neural cells (Figure 2I).

To cross-validate this assignment, we adapted Gruffi^27^ for detecting neural and non-neural transcriptomic signatures. We used GO terms for endoderm (GO:0001706) and mesoderm formation (GO:0001707) for selecting non-neural cells and GO terms for nervous system development (GO:0007399) and neurogenesis (GO:0022008) for selecting neural cells (Figure S4E). The results between reference-query mapping and Gruffi were mostly coherent (Figure S4F). Inconsistent annotations were observed for putatively muscular cells (positive for *MYOG* and *MYOD1*), which were incorrectly classified as neural by Gruffi. We suggest that this discrepancy may be due to the shared excitability between neural and muscular cells.

### Characterization of neural cell diversity

**Figure 3:**
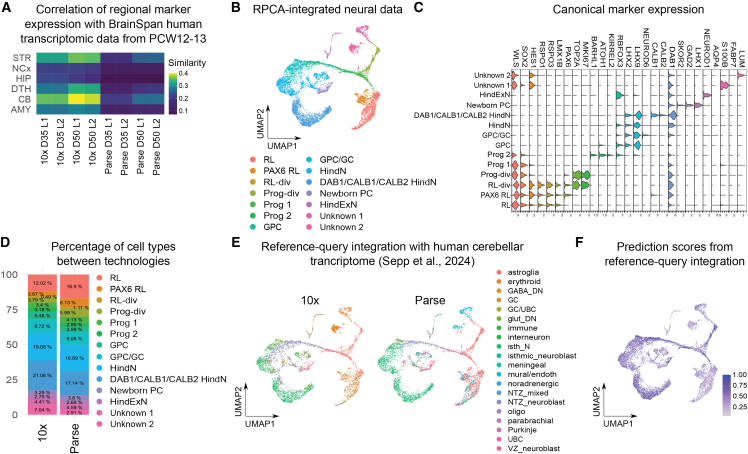
Assessment of regional identity and cell type annotation (A) Heatmap of similarity metric of VoxHunt algorithm comparing samples with human neocortical RNA-seq data from BrainSpan using brain regional markers obtained from Mouse Brain Atlas at E13 (B) UMAP plots for globally normalized and RPCA-integrated neural data with manually annotated clusters. (C) Violin plots for expression of canonical markers of hindbrain development. (D) Stacked bar plot representing average proportion of individual cell types between technologies. (E) UMAP plot representing cell type identity as assigned based on reference-query integration with human cerebellar transcriptome.^35^ (F) Feature plots showing prediction score based on reference-query integration with human cerebellar transcriptome. See also Figure S5 and Table S4.

Utilizing the results of reference-query mapping with the human developmental transcriptome,^26^ we subset neural cells (19,526 neural cells out of 37,452 cells) and downsampled 10x and Parse datasets to an equal number of cells (resulting in 7,212 cells per technology) before repeating integration and dimensionality reduction. Next, we aimed to reveal the brain regional identity of the neural cells within the cerebellar organoids^23^ by correlating regional marker gene expression (inferred from E15 mouse brain; Table S4) with our dataset and human brain transcriptomic data from postconceptional week (PCW) 12–13 from Brain Span.^33^^,^^34^ All samples had the highest correlation with the cerebellum (Figure S5A). However, when similarity scores were not scaled, they were higher for 10x than for Parse samples (Figure 3A). Next, we assigned cell identities to the neural cells by combining cerebellar canonical marker gene^18^^,^^35^^,^^36^^,^^37^ with differential gene expression (DGE). We identified both RL-derived cellular lineages (RL, granule precursor cells [GPC], and GC) and VZ-derived newborn PCs (Figures 3B and 3C). A subset of neuronal cells was characterized as hindbrain neurons (Figure 3B). While overall proportions of cells captured by the two technologies were similar (Figures 3D and S5B), dividing progenitors, PAX6-positive RL, and dividing RL cell populations were significantly enriched in Parse (Figures 3D and S5B). We then visualized the distribution of cell types in organoids originating from different cell lines (Figure S5C). This analysis revealed differences in proportions of different neural cell types between cell lines (Figure S5C). This highlights the necessity to use multiple cell lines and batches of differentiation when characterizing the reproducibility of new neural organoid protocols.

To our knowledge, currently scRNA-seq cerebellar organoid datasets are available for D60^21^ or D90^22^ of differentiation. We hypothesized that our scRNA-seq analysis at D35 and D50 provides insights into the establishment of cell type diversity during differentiation. Indeed, when we visualized the distribution of cell types between the two sampling time points, we found that RL-derived populations had higher proportions in D35 than in D50 of differentiation while several neuronal populations, including newborn PCs, demonstrated the opposite trend (Figures S5D and S5E). Therefore, cerebellar organoids recapitulated the temporal progression of cell type proportions characteristic of the developing cerebellum.^18^^,^^37^ To characterize the similarity of our cerebellar organoids with the developing human cerebellum, we performed reference-query mapping with a primary cerebellar transcriptomic dataset, subset to only include prenatal samples.^35^ While finding general agreement in cell type annotations, we noticed differences in both assigned cell type identities (Figure 3E) and prediction scores, which were higher in Parse than in 10x data (Figure S5F). We further compared our data with a recent scRNA-seq cerebellar organoids dataset (Figures S5G and S5H).^21^ The prediction scores were higher than for the comparison with the human cerebellar developmental transcriptome (Figure S5F). This time, however, prediction scores were higher for 10x than for Parse cells (Figure S5I). Interestingly, both reference datasets were generated using 10x. Therefore, expectedly, our organoid data aligns more with organoid data obtained from a different protocol than with primary tissue.

In summary, we found that the cerebellar organoids indeed acquired a mid-gestational human cerebellar regional identity. We also found robust differentiation into both major cerebellar lineages, RL- and VZ-derived cells. Small variances in the different parameters were found between 10x and Parse technologies.

Secondary analysis between techniques reveals differences in cell stress signatures and neurodevelopment-related gene regulatory networks activity.

**Figure 4:**
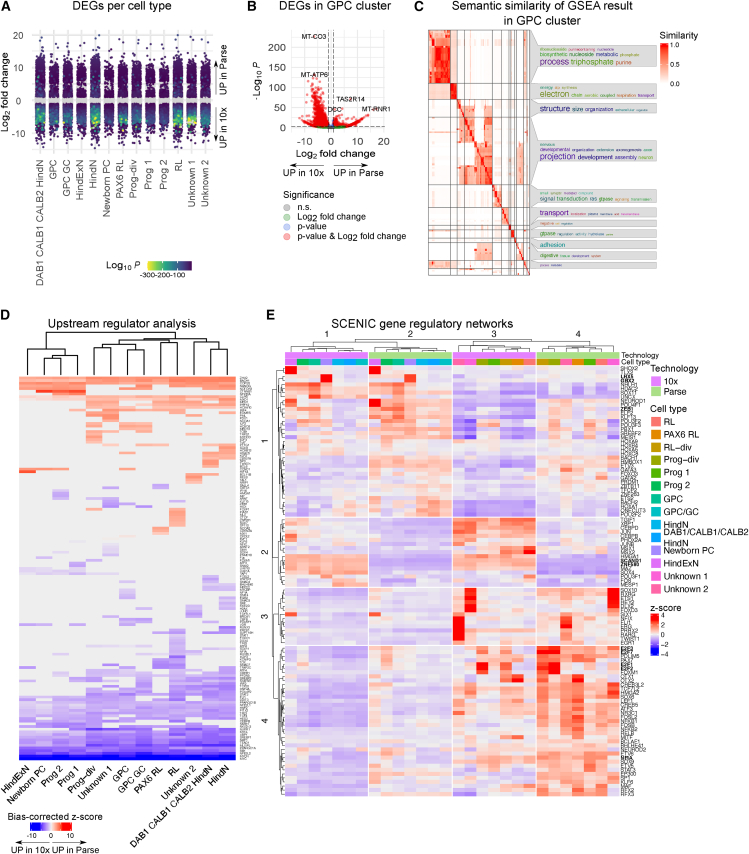
DGE between technologies (A) Strip plot displaying DEGs between technologies per cell type. Genes represented in gray are not differentially expressed. Color represents log10 adjusted *p* value for differentially expressed genes (absolute log2 fold change >1, FDR <10^−4^). (B) Volcano plot representing DGE in GPC cluster. (C) Heatmap representing semantic similarity between GO terms identified as significantly deregulated in GPC cluster by GSEA analysis. (D) Heatmap representing bias-corrected Z scores for upstream regulator analysis. (E) Heatmap representing *Z* scores for SCENIC regulon activity calculated based on AUC scores. See also Figure S6 and Tables S5 and S6.

During QC, we found differences in the percentage of reads originating from ribosomal and mitochondrial protein-coding genes between the two technologies (Figure 1B). We also found a subset of cells expressing cell stress-related genes, and this proportion was higher for 10x (Figure 2D). Therefore, we analyzed whether the neural cells preserved these transcriptomic features and performed DGE analysis between the different technologies within individual cell types. For that, we split the dataset by cell type, technology, cell line, and day of differentiation and pseudobulked cells for DESeq2. DEGs were spread across all cell types (Figures 4A and S6A). Especially mitochondrial and ribosomal protein-coding genes were upregulated in 10x compared to Parse (Table S5), including GPCs (Figure 4B). More genes were upregulated in 10x compared to Parse across all cell types (Figure S6A). Interestingly, there were a few genes with large fold change and relatively large *p* values upregulated in either of the two technologies (Figure S6B). To functionally characterize the differences in gene expression between the techniques, we performed gene set enrichment analysis (GSEA) and clustered the output in a semantic similarity matrix (Figure 4C). Here, we describe findings for GSEA in GPCs, as a representative cell type with relatively high cell numbers and a medium number of DEGs. In GPCs, the normalized expression score for all statistically significant GO terms was less than 0, indicating their upregulation in 10x compared with Parse (Table S6). One cluster of enriched GO terms was related to nucleotide processing, another to mitochondrial respiration. These two clusters of GO terms included not only mitochondria-encoded protein-coding genes (Figure 1B), but also nuclear-encoded genes involved in mitochondrial function (e.g., NDUF; Table S6). Another group of enriched GO terms in GPCs was described as related to neuron projection assembly (Figure 4C).

To reveal the upstream mechanisms leading to the transcriptional changes across cell types, we performed upstream regulator analysis (URA). That predicted a variety of TFs to be differentially active in either of the technologies, and that these transcriptional changes were coordinated across cell types (Figure 4D). For example, we found ER stress-induced TFs *XBP1*, *ATF4,* and *ATF6*, and *NFE2L2* and *NRF1*, which mediate oxidative stress response and are involved in maintaining mitochondria redox homeostasis^38^^,^^39^^,^^40^ to be upregulated in 10x. These predictions are in line with our previous findings (Figures 2B and S4A), demonstrating a higher proportion of stressed cells in 10x than Parse. Since we found that the Parse dataset had a larger proportion of reads originating from TFs (Figure 1B), we decided to extend our analysis to gene regulatory network (GRN) analysis using SCENIC.^41^ Average area under the curve (AUC) scores per cell type and technology were *Z* score normalized and subjected to *k-*means clustering (Figure 4E). We found that, albeit overall small *Z* scores, the two technologies clustered apart (column clusters 1 and 3 for 10x, and 2 and 4 for Parse) but also cell types divided into two meta groups based on the activity of GRNs (column clusters 1 and 2 were enriched in neurons, while column clusters 3 and 4 contained predominantly progenitor cell types; Figures 4E and S6C). Additional examples of cell type-associated regulon activity can be found in Figure S6C. In summary, transcriptional differences between technologies did not mask transcriptional differences between cell types.

## Discussion

In this study, we compared two broadly used and commercialized approaches for sample multiplexing of scRNA-seq: 10x and Parse using cerebellar organoids, as an example of a complex 3D sample that requires dissociation. Regionalized neural organoids, such as cerebellar organoids, are commonly used in neuroscience research but can be challenging due to heterogeneity between samples, batches, and iPSC lines and require in-depth characterization.^7^^,^^42^ To compare scRNA-seq datasets across experiments and to differentiate technical and biological causes of variance, it is essential to understand artifacts and biases introduced by experimental pipelines of cell capture techniques. We generated cerebellar organoids^23^ from three iPSC lines, dissociated the samples at D35 and D50, subjected them to 10x and Parse cell capture, and sequenced the resulting libraries. We compared the methods based on library efficiency, differential transcript capture, cell type enrichment, and secondary analysis insights.

Sample preparation differs considerably between the two technologies: Parse samples are fixed after dissociation, whereas cells are kept alive until lysis in 10x. Consequently, Parse provides more flexibility in sample processing and allows handling higher sample numbers in one sequencing run, which is advantageous for larger experiments. Further, we observed differences in the cell recovery rates—42.7% for 10x and 16.5% for Parse (Figure S3B), and for scarce samples, higher recovery is beneficial to maximize data output. The downside of fixation may be decreased RNA quality, reflected in a lower number of recovered genes.^43^ Next, there are substantial differences in library preparation protocols. Namely, one important difference is that Parse, in addition to oligo(dT) primers, uses random hexamer primers for reverse transcription, thus allowing multipriming. Since both technologies rely on reverse transcription of mRNA up to its 5′ end, multipriming may allow for more robust recovery of longer transcripts. Random hexamer primers also allow capturing transcripts devoid of polyA tails, non-coding RNAs, and nascent transcripts. Other steps of library preparation, albeit different, rely on reactions that should be either immune to transcript-specific biases or whose effect is difficult to predict.

For alignment and generation of count tables, we used technology-specific pipelines, thus leveraging optimal settings for both library construction protocols. Both approaches use the same tools for data processing with minor changes, thus potentially introducing minor variations into the resulting count tables.

Consistent with previous findings^6^ and suggested effects of different sample processing and library generation protocols, we observed differences in the number of detected genes and their properties. Namely, 10x resulted in a higher number of genes, a higher number of protein-coding genes, including mitochondrial and ribosomal protein-coding genes compared to Parse (Figures 1B and S3E). Furthermore, 10x captured transcripts with higher GC content, while Parse captured longer transcripts (Figures 1B and S3E–S3H). Previous studies showed a connection between gene length and neurodevelopment and NDDs.^44^^,^^45^ Interestingly, BCL11b (CTIP2) (102,911 bps), a TF crucial for neuronal maturation and differentiation, is predicted to be upregulated in Parse in DAB1/CALB1/CALB2 HindN in our data (Figure 4D). The clinical features of BCL11b-associated NDDs include ASD, intellectual disability, and cerebellar hypoplasia,^45^ which have been previously modeled in organoids.^8^^,^^9^ These findings highlight transcript length as an important factor, suggesting Parse may be better suited for studying long transcripts upon experimental manipulations.

Further, Parse covered more transcripts encoding TFs among protein-coding genes (Figures 1B and S3E). To investigate if this bias had effects on GRN activity, we employed SCENIC analysis. Interestingly, Parse showed higher *Z* scores for neurodevelopment and maturation-related regulons (Figure 4E), in contrast to the upregulation of neuron processes assembly-related terms in 10x in GSEA (Figure 4C). Additionally, we identified cell type- and technique-specific differences in regulon activity. For instance, *NFIA* regulon had higher *Z* scores in RL derivates in Parse (Figure 4E), a TF relevant for GC maturation and linked to NDDs and gliomas.^46^^,^^47^ Taken together, the GRN analysis reveals not only cell type but also technique-driven differences in regulon activity of identical biological samples.

During QC, we found that the percentage of mitochondrial and ribosomal protein-coding genes was higher in 10x samples (Figure 1B), corroborating previous findings.^6^ DGE analysis revealed the upregulation of mitochondrial protein-coding genes and other genes involved in mitochondrial function (Figure 4B). Hence, the differences in mitochondrial transcripts might be partially explained by higher cell stress in the 10x data and mitochondrial involvement in stress response pathways.^48^

We analyzed stress-specific modulators and identified three modules (oxidative stress, glycolysis, and ISR) that separated the two technologies. 10x showed stronger stress module expression overall, particularly at D50. Regionalized neural organoids have been reported to show high expression of stress pathway-related transcripts due to *in vitro* culturing conditions and insufficient oxygen supply.^27^^,^^28^^,^^29^^,^^49^ The cascade of events unfolding upon persistent hypoxia may explain the elevated stress response-associated transcriptional signature at D50 compared to D35 of differentiation (Figure 2B).^50^ Additionally, tissue dissociation for single-cell capture can induce stress response.^51^ Since stressed cells are common in scRNA-seq organoid datasets, a bioinformatic approach called Gruffi was developed to remove these cells.^27^ Using Gruffi, we found a noticeably higher percentage of stressed cells in the 10x compared to the Parse dataset at both time points (Figure 2D). Immediate fixation of cells after the dissociation in Parse may limit the induction of stress-related genes, in contrast to live cells processing in 10x. These findings suggest that identical samples of cerebellar organoids show a technology- and time point-specific stress response reflected in transcriptional signature and striking differences in the number of cells identified as stressed (Figure 2D).

To assess the biological reproducibility of organoid differentiation, we assessed the percentage of neural cells,^26^ revealing neural commitment of 52.1% of all cells, suggesting the initial tissue specification could be improved. Different neural organoid protocols^52^^,^^53^ and a recently published protocol for cerebellar organoids^21^ use dual SMAD inhibition during initiation of differentiation to prevent meso- and endodermal fates thus promoting neural induction.^54^ In contrast, the cerebellar differentiation protocol used in this study employs only one SMAD pathway inhibitor,^23^ and dual SMAD inhibition could improve neuroectodermal commitment. Furthermore, we noticed substantial differences in differentiation efficiencies between the iPSC lines, with the KOLF2.1J-derived cerebellar organoids demonstrating the lowest proportion of neural cells. This suggests that iPSC line-inherent mechanisms influence the differentiation efficiency,^55^ underscoring the importance of using isogenic control iPSCs when analyzing pathogenic variants.^56^ A recent study suggests adjusting small molecule and growth factor concentrations in cortical organoids for individual iPSC lines to reduce off-target tissue.^40^ This approach could reduce line-to-line variability. Further, a recent preprint demonstrates structural variants in neurodevelopmental genes in KOLF2.1 line that could affect neural differentiation.^57^ Despite the differences between the three iPSC lines used in this study, our cerebellar organoids generated cerebellar cells of both RL and VZ lineage. Comparing our dataset with a recently published cerebellar organoid transcriptomic dataset^21^ revealed similar cell populations.

In conclusion, our comparison of Parse and 10x encompassed library efficiency, differential transcript capture, cell type preferences, and secondary analysis outcomes, showing distinct strengths of each method. While 10x provided higher cell recovery and gene detection rates, Parse captured longer transcripts and a wider range of transcript lengths and resulted in lower cell stress—important for regionalized neural organoids, in which cell stress may be a key artifact.^28^ These technical differences have relevant biological implications, making it essential to choose the appropriate method based on specific research goals. Future studies should consider these factors to improve the accuracy and biological relevance of single-cell transcriptomic analyses. Finally, we demonstrated cerebellar organoid differentiation and in-depth characterization on three iPSC lines, highlighting the importance of using diverse cell lines to capture line-to-line variability.

### Limitations of the study

In the current study, we used cerebellar organoids as a model system to showcase technical differences arising from two single-cell capturing and multiplexing techniques. Our experimental design has several limitations. First, the study relied on one differentiation per line, reducing our ability to separate protocol effects from batch- and line-specific variability. Next, although the organoids produced relevant cerebellar lineages, neural commitment was incomplete, and a substantial fraction of cells adopted non-neural fates, indicating that early tissue specification was not fully optimized. More recent cerebellar organoid differentiation protocols^21^^,^^58^^,^^59^ may better suppress meso-/endodermal trajectories and improve neural induction through dual SMAD inhibition, and should be evaluated in future work. In addition, differentiation efficiency differed markedly across cell lines, with the KOLF2.1J line showing the lowest neural yield, suggesting that line-inherent properties may confound direct comparisons.^57^^,^^60^ Finally, some technical features of the single-cell workflows may introduce differences in count tables due to bioinformatic processing. In our analysis, we generated count tables using technology-specific pipelines, ensuring optimal settings. While most of the bioinformatic tools are shared between pipelines, some minor differences exist. For example, while both Cell Ranger and split-pipe perform UMI demultiplexing, only Cell Ranger reports further UMI correction and exclusion of low-quality UMIs.

## Resource availability

### Lead contact

Further information and requests for resources and reagents should be directed to and will be fulfilled by the lead contact, Simone Mayer (simone.mayer@kit.edu).

### Materials availability

This study did not generate new unique reagents or new iPSC cell lines.

### Data and code availability

• Single-cell RNA sequencing data are available at CellXGene (link: https://cellxgene.cziscience.com/collections/0dd101f7-9829-44b3-a323-18b113eabeb4).
• This study did not generate novel code, and the required functions for the analysis and data visualization are described in the STAR Methods section.
• Further requests for additional information should be directed to the lead contact, Simone Mayer (simone.mayer@kit.edumailto).

## Acknowledgments

We thank Antje Schulze-Selting, Elisabeth Gustafsson, Christina Kulka, and Ezgi Atay for technical support. We thank Christopher Sifuentes, Yogesh Singh, and Vincent Hammer for strategic and technical discussions. We are grateful for financial support from the Hertie Foundation (10.13039/501100003493Gemeinnützige Hertie-Stiftung), the 10.13039/100011937Ministerium für Wissenschaft, 10.13039/501100003542Forschung und Kunst Baden-Württemberg state postgraduate fellowship (to T.K.), Add-on Fellowship of the 10.13039/100008662Joachim Herz Foundation (to K.S.), and the 10.13039/100008661Heidelberger Akademie der Wissenschaften (WIN Kolleg). NGS sequencing for the Parse libraries was performed with the support of the 10.13039/501100001659DFG-funded 10.13039/100015863NGS Competence Center Tübingen (INST 37/1049-1). This project has been made possible in part by grant number 2022-316727 from the Chan Zuckerberg Initiative DAF, an advised fund of 10.13039/100000923Silicon Valley Community Foundation. This research has been partially funded by the 10.13039/501100001659Deutsche Forschungsgemeinschaft (DFG, German Research Foundation) under Germany’s Excellence Strategy via the Excellence Cluster 3D Matter Made to Order (EXC-2082/1-390761711).

## Author contributions

K.S., conceptualization, methodology, software, formal analysis, writing – original draft, writing – review and editing, visualization, and project administration; T.K., conceptualization, methodology, investigation, writing – original draft, writing – review and editing, visualization, and project administration; V.L., methodology, software, formal analysis, writing – original draft; writing – review and editing; F.C., investigation, writing – original draft; writing – review and editing; Z.Y., investigation, writing – original draft; writing – review and editing; K.B., investigation; writing – review and editing; J.M., funding acquisition; writing – review and editing; N.C., conceptualization, methodology, formal analysis, writing – original draft, writing – review and editing, resources, and supervision; S.M., conceptualization, methodology, writing – review and editing, resources, supervision, and funding acquisition.

## Declaration of interests

K.S. is currently affiliated with Oncode Institute, Hubrecht Institute, KNAW, and University Medical Center Utrecht, Utrecht, the Netherlands. V.L. is currently affiliated with the Technical University of Munich, Munich, Germany.

## Declaration of generative AI and AI-assisted technologies in the writing process

During the preparation of this work, the authors used ChatGPT in order to improve the language and readability. After using these tools, the authors reviewed and edited the content as needed and take full responsibility for the content of the publication.

## STAR★Methods

### Key resources table

**Table:** 

REAGENT or RESOURCE	SOURCE	IDENTIFIER
**Antibodies**		

CORL2 (SKOR2)	Atlas Antibodies	Cat# HPA046206; RRID: AB_2679588
Sox2	R&D Systems	Cat# AF2018; RRID: AB_355110
BARHL1	Atlas Antibodies	Cat# HPA004809; RRID: AB_1078266
ATOH1	Sigma-Aldrich	Cat# WH0000474M1; RRID: AB_1839957
Ki67	Merck	Cat# AB9260; RRID: AB_2142366
Tuj1 (TUBB3)	Atlas Antibodies	Cat# AMAb91394; RRID: AB_2716670
Map2	Abcam	Cat# ab32454; RRID: AB_776174

**Critical commercial assays**		

Chromium Next GEM Single Cell 3′ kit v3.1	10x Genomics	Cat# 1000268
Evercode WT Mini v2	Parse Biosciences	Cat# ECW02110

**Deposited data**		

scRNA-seq data of hiPSC-derived cerebellar organoids	This paper	CZ CELLxGENE: https://cellxgene.cziscience.com/collections/0dd101f7-9829-44b3-a323-18b113eabeb4
Human fetal development scRNA-seq	Cao et al.^26^	https://doi.org/10.1126/science.aba7721
BrainSpan human developmental transcriptome	Miller et al.^34^	https://doi.org/10.1038/nature13185
Human cerebellar development scRNA-seq	Sepp et al.^35^	https://doi.org/10.1038/s41586-023-06884-x
hiPSC-dericed cerebellar organoids scRNA-seq	Atamian et al.^21^	https://doi.org/10.1016/j.stem.2023.11.013

**Experimental models: Cell lines**		

BIONi010-C (male)	EBiSC	hiPSC
BIONi037-A (female)	EBiSC	hiPSC
KOLF2.1J (male)	Jackson Laboratory	hiPSC

**Software and algorithms**		

Cell Ranger v.7.2.0	10x Genomics	https://www.10xgenomics.com/support/software/cell-ranger/downloads
Split-pipe v.1.1.2	Parse Biosciences	https://www.parsebiosciences.com/

### Experimental model and study participant details

#### iPSC culture

Commercially available iPSC lines BIONi010-C (EBiSC), BIONi037-A (EBiSC) and KOLF2.1J (Jackson Laboratory) were cultured under standard conditions (37°C, 5% CO2, and 100% humidity) in E8 Flex medium (BIONi010-C and BIONi037-A, Gibco, Cat. no. A2858501) and mTeSR plus (KOLF2.1J, STEMCELL Technologies, Cat. no 100–0276) on hESC-qualified growth factor-reduced Matrigel-coated (Corning, Cat. no. 354277) cell culture dishes (Greiner, Cat. no. 657160). Passaging was performed using Gentle Dissociation Reagent (STEMCELL Technologies, Cat. no. 07174) once cells reached 80%–90% confluency. Medium was supplemented with Thiazovivin (Sigma-Aldrich, Cat. no. 420220) upon passaging for one day. All cell lines were kept under passage 20 and tested for mycoplasma using PCR Mycoplasma Detection Set (TaKaRa, Cat. no. 6601) and pluripotency by immunocytochemistry against OCT4 (rabbit, 1:500, Abcam, Cat. no. ab19857).

#### Generation of cerebellar organoids

Cerebellar organoids were generated as previously described^23^^,^^61^ with some modifications: 80–90% confluent iPSCs were dissociated using Accutase (Merck, Cat. no. A6964), and 4,500 cells per well were seeded into 96 well plates (S-bio, Cat. no. MS-9096VZ) in culture medium (Gibco, Cat. no. A2858501), supplemented with 10 μM Y-27632 (Cayman Chemical, Cat. no. 10005583). Once aggregates reached 250 μm in diameter, medium was changed to growth factor-free chemically defined medium (gfCDM) supplemented with 50 ng/mL FGF2 (PeproTech, Cat. no. 100-18B) and 10 μM SB-431542 (Tocris, Cat. No. 1614). At D7, FGF2 and SB-431542 were reduced to 33.3 ng/mL and 6.67 μM, respectively. At D14, media was supplemented with 100 ng/mL FGF19 (PeproTech, Cat. No. 100-32). The medium was changed to Neurobasal Medium at D21, supplemented with 300 ng/mL SDF-1 from D28 to D34. From D35 onwards, media was changed to complete BrainPhys (StemCell Technologies, Cat. no. 5793), supplemented with 10 μg/mL BDNF (PeproTech, Cat. no. 450-02), 100 μg/mL GDNF (PeproTech, Cat. no. 450-10), 100 mg/mL dbcAMP (PeproTech, Cat. no. 1698950) and 250 mM ascorbic acid (Tocris, Cat. no. 4055). All three cell lines were processed in parallel throughout the experiments.

### Method details

#### Immunohistochemistry on organoids

Organoids were fixed at the respective time points in 4% PFA in PBS for 45–60 min at room temperature.^62^ The organoids were washed three times for 15 min with 1× PBS and then incubated in 30% sucrose (Sigma-Aldrich, S7903) in PBS solution at 4°C until they sunk to the bottom of the dish. The organoids were embedded in a 1:1 v/v mixture of 30% sucrose in PBS and optimal cutting temperature (OCT) compound (Sakura, 4583) and sectioned on Superfrost Plus slides (R. Langenbrinck GmbH, 03–0060) with a cryostat at 20 μm (Leica). The slides were stored at −80°C.

For immunohistochemistry, slides were thawed for 15 min at room temperature, and the embedding solution was rinsed off with PBS. A hydrophobic pen (PAP pen, Abcam, ab2601) was used to circle the sections to prevent the blocking solution from spilling during incubation. Permeabilization and blocking were performed with 1% Triton X-100, 0.2% gelatin (Sigma-Aldrich, G1890) and 10% normal donkey serum in PBS for 1 h at room temperature. Primary antibodies were diluted in permeabilization and blocking solution and applied to the sections overnight at 4°C. Subsequently, the slides were rinsed with PBS three times for 15 min, then secondary antibodies were diluted in permeabilization and blocking solution and applied for 3 h at room temperature. The sections were rinsed in PBS three times for 15 min and nuclei were stained with DAPI (1:5000) diluted in PBS for 4 min. The sections were then rinsed in PBS and mounted using ProLong Gold (Invitrogen, P36930). Image acquisition was performed at 20X magnification using ECHO Revolution Hybrid Automated Microscope (DISCOVER ECHO INC.).

#### Single-cell dissociation of cerebellar organoids, library preparation, and sequencing

On D35 and D50, 24 organoids per cell line were pooled and dissociated using the Papain dissociation kit (Worthington, Cat.No. LK003150) following a published protocol with minor modifications.^52^ Cells were counted and divided into aliquots for further processing.

For the 10x Genomics pipeline cells were labeled with cell multiplexing oligos (CMO, 10x Genomics, Cat. no. 1000261) and subsequently pooled at an equal ratio. Cells were counted and loaded onto two lanes of a Chromium Next Gen Chip G (10x Genomics, Cat. no. 1000120) with a targeted cell recovery of 12,000 (D35) and 14,000 (D50) cells per lane. Library preparation was performed with the Chromium Next GEM Single Cell 3′ kit v3.1 (10x Genomics, Cat. no. 1000268), and sequencing was performed on NovaSeq 6000 with S1 flow cell kit and 100 cycles (Illumina, Cat. no. 20028319).

Samples for Parse Bioscience workflow were fixed using the Evercode fixation kit for cells (Parse Bioscience, Cat. No. WF300). Fixed samples were stored at −80°C. Samples were characterized by day of differentiation (D35 or D50) and cell line (BIONi010-C, BIONi037-A, or KOLF2.1J). Every sample was loaded as a technical duplicate into 2 independent wells, with all samples spanning wells 1–12. Sequencing was performed using a molarity of 62.4 nM and 3% PhiX spike-in on the Nova Seq 6000 with SP flow cell kit and 200 cycles (Illumina).

### Quantification and statistical analysis

#### Data downsampling, preprocessing, and quality control

Initially, the datasets from 10x and Parse pipelines had different sequencing depths (Table S1). To ensure fair comparison, we downsampled both datasets to an average of 50,000 reads per cell. The FASTQ files were downsampled with the *seqtk sample* tool using the same seed for forward and reverse reads. Parse FASTQ files from each of the 2 sub-libraries were demultiplexed into 6 samples and processed *split-pipe* (v1.1.2), resulting in a count matrix. The 2 sub-libraries were merged with *combine* mode of *split-pipe*. For 10x data, read downsampling was performed for individual libraries. Afterward, downsampled FASTQ files were processed with *cellranger* (v.7.2.0) *multi* pipeline, assigning their cell line of origin based on CMO.

Gene names in count matrices between the two technologies were harmonized as follows: First, ENSEMBL gene identifiers were used to merge expression matrices. Secondly, ENSEMBL identifiers were replaced by HGCN identifiers wherever possible (41,980 genes), and ENSEMBL identifiers were used in other cases (20,930 genes). The merged count matrix was converted into a Seurat object (Seurat v.5.1.0). Gene biotypes were retrieved from bioMart using ENSEMBL identifiers. Ribosomal and mitochondrial protein-coding genes were identified by HGCN names starting with RPS/RPL and MT-, respectively. The percentage of gene expression for ribosomal and mitochondrial protein-coding genes as well as for individual gene biotypes were calculated using *PercentageFeatureSet()*. For transcription factors (TF) among protein-coding genes, the count matrix was first subset to protein-coding genes, and *PercentageFeatureSet()* was applied using the human TFs list.^63^

Next, QC was performed on cell and gene levels. Cells were excluded if they met any of the following criteria: (1) number of genes per cell ≤2,000 or ≥13,000; (2) number of genes per UMI ≤0.8; or (3) percentage of mitochondrial genes ≥8%. Genes were excluded if their cumulative expression across all cells was ≤8.

#### Data normalization, clustering, integration, and dimensionality reduction

After QC, data were normalized using Seurat’s *NormalizeData()* function with default parameters. Normalized data were then scaled, and principal component analysis (PCA) was performed on the z-scaled expression of the 2,000 most variable features (*FindVariableFeatures()*). Additionally, normalized counts were integrated using *IntegrateData()* function with reciprocal PCA (RPCA). Dimensionality reduction and clustering were performed using both un- and integrated data. *RunUMAP()* function was used for dimensionality reduction with 30 neighbors and 30 principal components (PC). Louvain clustering was performed using *FindClusters()* function.

#### Technology-specific analyses: Correlation analysis, transcript length, and GC content

To analyze the correlation of gene expression between technologies, we used cells that passed QC, averaged the gene expression for each technology, and calculated Pearson’s correlation coefficient. DEGs between technologies were identified using the MAST algorithm in *FindMarkers()* function as previously described^6^ with the following cutoffs: absolute log2 fold change (log2FC) > 1, adjusted *p*-value <0.01. Gene length and GC content were retrieved from bioMart.

#### Cellular stress assessment

Normalized unintegrated counts were used to analyze the expression of cell stress-related GO terms using *AddModuleScore()* function. A random set of genes with mean GO term size was used as an internal control for module expression analysis. Hierarchical clustering was performed on mean module expression of cell stress-related GO terms across samples. Gruffi cell stress analysis was performed using normalized unintegrated counts following the authors’ instructions.^27^ Two GO terms were chosen for negative selection: glycolytic process (GO:0006096) and integrated stress response signaling (GO:0140467); and one for positive selection: neurogenesis (GO:0022008).

#### Germ layer assessment

Normalized integrated counts were used to perform Azimuth reference-query mapping^30^ of our dataset with human fetal development transcriptome.^26^ Cells were further classified as “neural” and “non-neural” based on cell type assignment from Azimuth (Table S3). Gruffi differentiation lineage analysis was performed using normalized integrated counts. Two GO terms were chosen for negative selection: endoderm (GO:0001706) and mesoderm (GO:0001707) formation; and two for positive selection: nervous system development (GO:0007399) and neurogenesis (GO:0022008).

#### Neural data processing and cell type annotation

After germ layer assessment, the dataset was subset to neural cells by labels originating from Azimuth reference-query mapping and downsampled to retain the equal number of cells in 10x and Parse datasets (7,212 cells per technology). Data normalization, clustering, integration, and dimensionality reduction workflow steps were repeated as previously described.

VoxHunt^33^ was used to analyze the brain region identity of cells. 10 genes with the highest AUC scores per brain region in the developing mouse brain at E15 were retrieved, resulting in 186 unique regional marker genes. These marker genes were used to assess the similarity of gene expression profiles between our samples and BrainSpan human developmental transcriptome^34^ at postconceptional weeks 12 and 13.

Cell type annotation was performed for clusters at resolution 0.9 by a combination of approaches: (1) retrieving cluster marker genes by *FindAllMarkers()* with MAST (normalized counts provided as input) and ROC (raw counts provided as input) algorithms; (2) visualizing canonical marker gene expression for cell types in the developing mouse and human cerebellum.

#### Reference-query mapping with published primary cerebellar development and cerebellar organoids transcriptomic datasets

For reference-query mapping of neural-classified cells, we first used human cerebellar development transcriptomic dataset^35^ as a reference, downsampling it to 1,000 cells per cell type as defined by the metadata (author_cell_type column). Both the reference and query datasets were normalized, variable features identified, scaled, and PCA was performed using Seurat’s default parameters. Integration was performed using *FindTransferAnchors()* function with the “pcaproject” option and 30 PCs. Predicted cell types and prediction scores were obtained from *TransferData(),* wrapped into *MapQuery()*, with default parameters and “author_cell_type” as the reference label. For integration with the cerebellar organoids transcriptomic dataset,^21^ the same method was used with two adjustments: (1) the complete reference dataset was used for mapping; (2) the reference label was “final.clusters”.

#### Differential gene expression analysis and functional enrichment analysis

For DGE analysis, raw counts from neural cells were used. Cells were grouped by cell type, technology, cell line, and day of differentiation, excluding groups with fewer than 20 cells. Gene counts were aggregated by technology, cell line, and day of differentiation using *AggregateExpression()* function with default settings to sum raw counts per group. No further downsampling was applied to equalize cell group sizes. The aggregated counts were used for *DESeq2* (v.1.42.1) DGE analysis between technologies within individual cell types.^64^ Log2FC were shrunk using *apeglm* shrinkageestimator.^65^ Volcano plots were generated using *EnhancedVolcano* library (v.1.20.0).

GSEA with GO terms was performed by *clusterProfiler* (v.4.10.1)^66^ using Biological Processes gene ontology, gene set size of 50–500 genes, false discovery rate (FDR) for *p*-value adjustment with a q-value threshold of 0.05. For significantly deregulated GO terms, similarity matrices were calculated and simplified using the *binary cut* approach implemented in *simplifyEnrichment* (v.1.12.0) package.^67^

#### Upstream regulator analysis

Upstream regulator analysis was conducted using IPA software (Qiagen). Cell type-specific DESeq2 output matrices were used for IPA core analysis with the following cutoffs: (1) absolute log2FC > 1; (2) q-value <0.0001. For visualizations, molecule type was restricted to transcription regulators, and bias-corrected z-scores across cell types were used for hierarchical clustering using the *ComplexHeatmap* package (v.2.18.0). When z-scores were not available, they were set to 0.

#### Gene regulatory network activity analysis

We performed GRN analysis closely following the official pySCENIC protocol.^41^^,^^68^ The annotated raw count matrix from Seurat and the list of human TFs were processed, inferring importance values of regulatory interactions between TFs and their target genes. The inferred interactions (“adjacencies”) were searched in the cisTarget databases to identify enriched binding motifs. TFs and target genes indicated by the enriched motifs were grouped into regulons, and their enrichment was assessed in each cell. Cells were assigned AUC scores representing activity levels of regulons. Z-scores were calculated based on AUC scores, and k-means clustering of z-scores was performed to reveal groups of co-regulated regulons. Regulon target genes and GO BP were used for gene set overrepresentation analysis (ORA) by clusterProfiler (v.4.10.1) with gene set size of 5–500 genes, FDR for *p*-value adjustment method, and a q-value threshold of 0.1.

#### Statistics

R v.4.3.2 was used for statistical analysis. Statistical tests are described in text and figure legends, and results are documented in Table S7. Two-sided unpaired t-tests were used to compare two groups. For comparisons with more than two groups, we used three-way ANOVA. Within a set of comparisons (e.g., for quality control metrics), the Benjamini-Hochberg method of *p*-value adjustments was used.

## Supplemental information

Document S1. Figures S1–S6

Table S1. Sequencing statistics, related to Figure 1

Table S2. Summary of sequencing statistics, related to Figure 1

Table S3. Assignment of neural vs. non-neural cell fate to the human developmental transcriptome dataset, related to Figure 2

Table S4. Cerebellar regional marker genes, related to Figure 3

Table S5. Differentially expressed genes between 10x and Parse, related to Figure 4

Table S6. GSEA results for GPC cluster, related to Figure 4

Table S7. Detailed statistical results related to Figures 1, 3, S3, and S5
