# Supplementary material for: Comparing the impact of sample multiplexing approaches for single-cell RNA-sequencing on downstream analysis using cerebellar organoids
Source: iScience. 2026 Jan 22;29(2):114780. doi: 10.1016/j.isci.2026.114780 (PMC12930056; doi:10.1016/j.isci.2026.114780)
Supplement: Document S1. Figures S1–S6 [file mmc1.pdf]

iScience, Volume *29*

Supplemental information

Comparing the impact of sample multiplexing approaches for single-cell RNA-sequencing on downstream analysis using cerebellar organoids

Kseniia Sarieva, Theresa Kagermeier, Vladislav Lysenkov, Francesco Castagnetti, Zeynep Yentuer, Katharina Becker, Julia Matilainen, Nicolas Casadei, and Simone Mayer

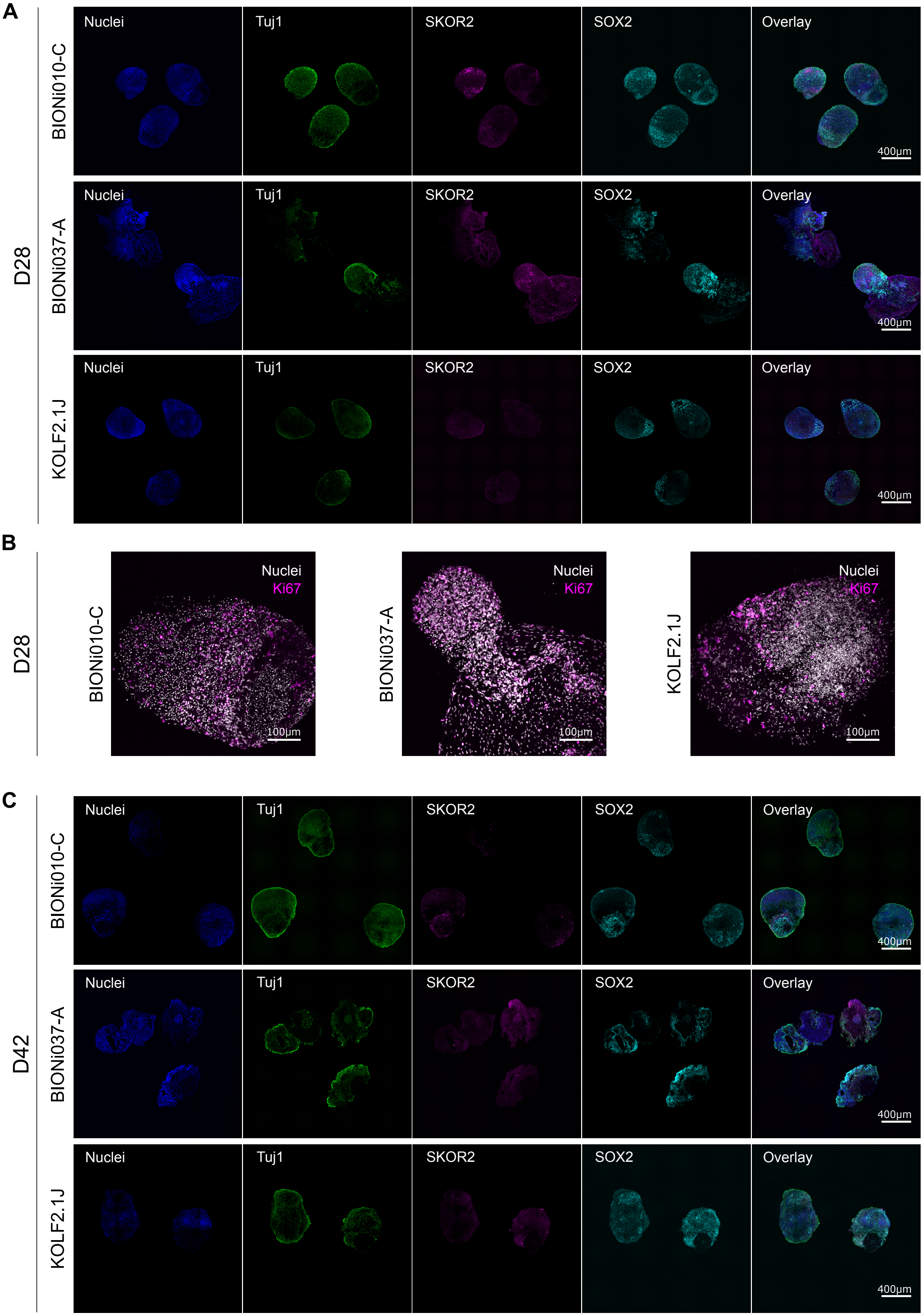

A
D28
BIONi010-C
BIONi037-A
KOLF2.1J
Nuclei
Tuj1
SKOR2
SOX2
Overlay
400µm
B
D28
BIONi010-C
BIONi037-A
KOLF2.1J
Nuclei
Ki67
100µm
C
D42
BIONi010-C
BIONi037-A
KOLF2.1J
Nuclei
Tuj1
SKOR2
SOX2
Overlay
400µm

Fig. S1. Immunohistochemistry of BIONi010-C, BIONi037-A, and KOLF2.1J cerebellar organoid sections at D28 and D42 of differentiation into the cerebellar lineage. **A-C**, Expression of the early neuronal marker Tuj1 (green), the neural progenitor marker SOX2 (cyan), and the excitatory granule cell progenitor marker SKOR2 (magenta) in D28 (**A**) and D42 (**C**) cerebellar organoids (representative images show organoids derived from BIONi010-C, BIONi037-A, and KOLF2.1J lines). Scale bars, 400 µm. **B**, Confocal images of immunohistochemistry against Ki-67 (magenta) in cerebellar organoids at D28 of differentiation derived from BIONi010-C, BIONi037-A, and KOLF2.1J lines. Scale bars, 100 µm.

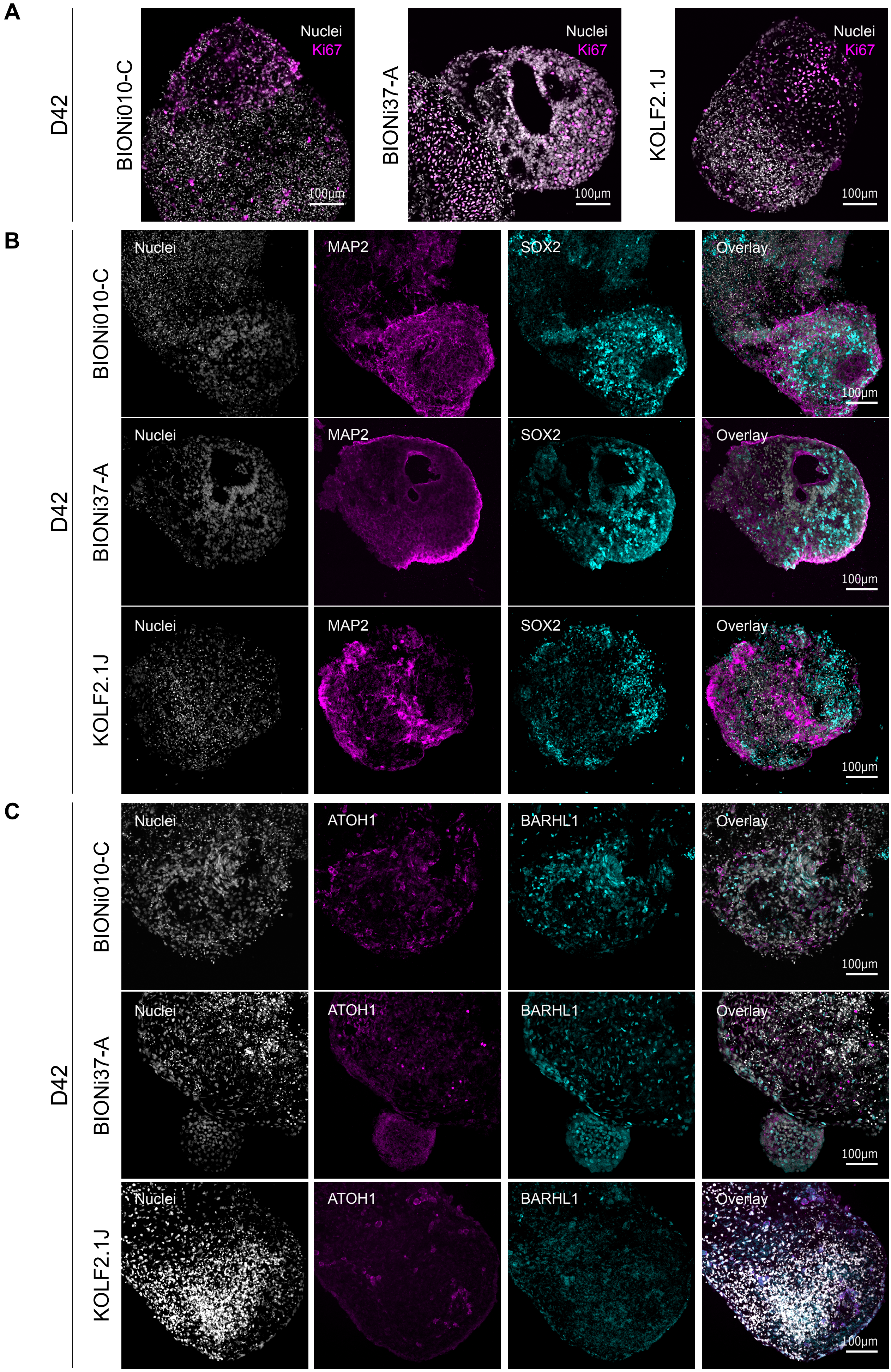
A
D42
BIONi010-C
BIONi37-A
KOLF2.1J
Nuclei
Ki67
100µm
B
MAP2
SOX2
Overlay
C
ATOH1
BARHL1

Fig. S2. Immunohistochemistry of BIONi010-C, BIONi037-A, and KOLF2.1J cerebellar organoid sections at D42 of differentiation into the cerebellar lineage. **A**, Confocal images of immunohistochemistry against Ki-67 (magenta) in cerebellar organoids at D42 of differentiation derived from BIONi010-C, BIONi037-A, and KOLF2.1J lines. Scale bars, 100 µm. B,C, Cerebellar organoids show the neuronal marker MAP2 (magenta), the neural progenitor marker SOX2 (cyan) (**B**); the rhombic lip progenitors ATOH1 (magenta) and the granule cell progenitors BARHL1 (cyan) (**C**) at D42 of differentiation (representative images show organoids derived from BIONi010-C, BIONi037-A, and KOLF2.1J lines). Scale bars, 100 µm.

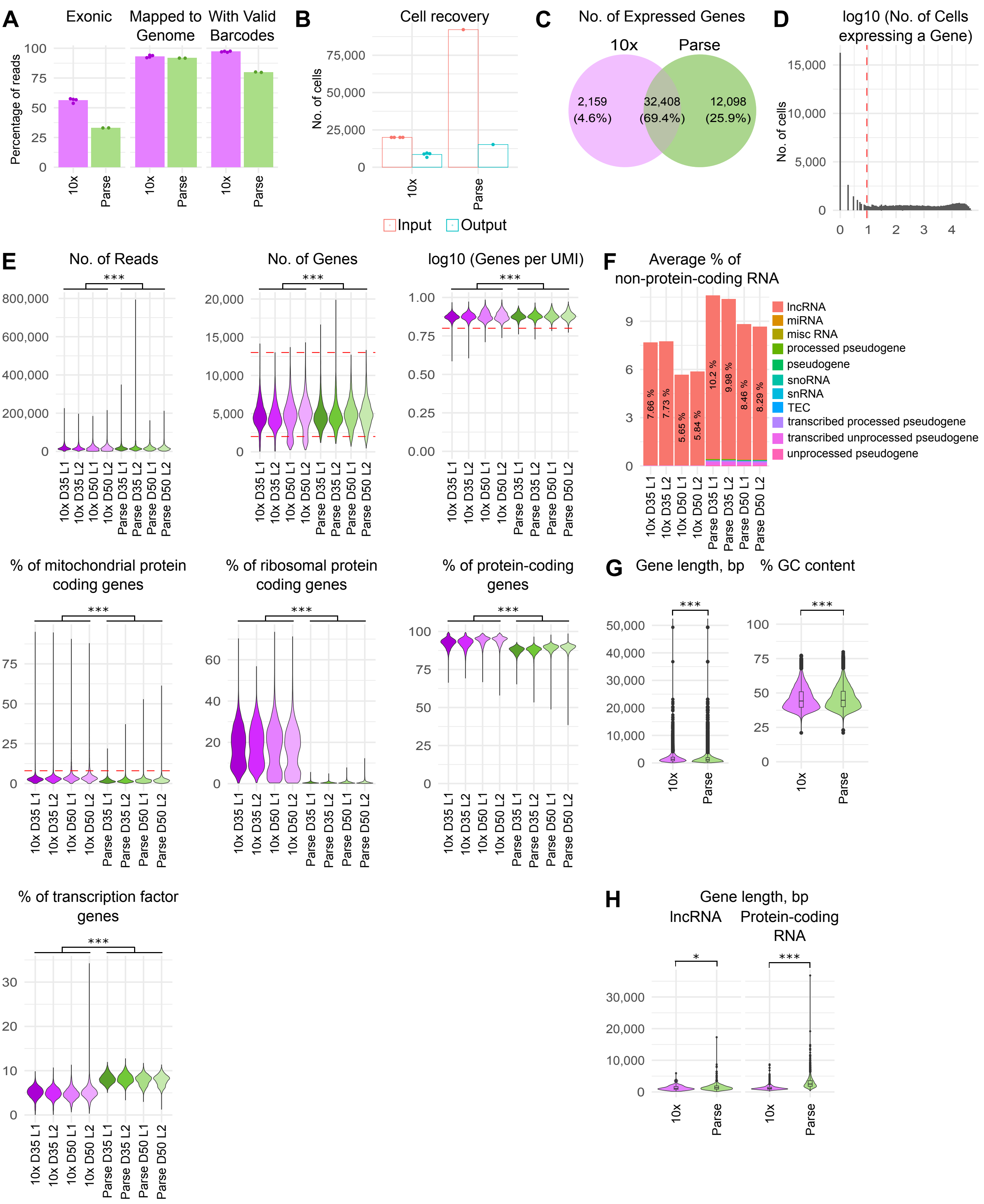
A
Exonic
Mapped to Genome
With Valid Barcodes
Percentage of reads
10x
Parse
B
Cell recovery
No. of cells
Input
Output
C
No. of Expressed Genes
10x
Parse
2,159 (4.6%)
32,408 (69.4%)
12,098 (25.9%)
D
log10 (No. of Cells expressing a Gene)
No. of cells
E
No. of Reads
No. of Genes
log10 (Genes per UMI)
% of mitochondrial protein coding genes
% of ribosomal protein coding genes
% of protein-coding genes
% of transcription factor genes
10x D35 L1
10x D35 L2
10x D50 L1
10x D50 L2
Parse D35 L1
Parse D35 L2
Parse D50 L1
Parse D50 L2
F
Average % of non-protein-coding RNA
7.66 %
7.73 %
5.65 %
5.84 %
10.2 %
9.98 %
8.46 %
8.29 %
lncRNA
miRNA
misc RNA
processed pseudogene
pseudogene
snoRNA
snRNA
TEC
transcribed processed pseudogene
transcribed unprocessed pseudogene
unprocessed pseudogene
G
Gene length, bp
% GC content
H
Gene length, bp
lncRNA
Protein-coding RNA

Fig. S3. Complementary to Fig. 1. Quality control and gene quantification biases in the data. **A**, Percentage of raw reads mapping to exonic regions, genome, and having valid barcodes. Bars represent the mean; dots represent the individual libraries. **B**, Numbers of input and output cells. Bars represent the mean; for 10x data, dots represent individual libraries; for Parse data, dots represent the total number of cells in the experiment. **C**, Venn diagram of genes expressed in at least 1 cell in each of the two technologies. Color represents technology. **D**, Distribution of the number of cells expressing a gene. **E**, Quality statistics before quality control. Red dashed lines represented threshold values. Color represents sample identity with respect to technology (10x or Parse), day of differentiation (D35 or D50), and library (L1 or L2). 10x, n = 33,951, Parse, n = 15,226 cells. Three-way ANOVA, p-values represent differences between technologies, *** $p < 0.001$. **F**, Stacked bar plot representing average proportion of reads originating from non-protein-coding RNAs (ncRNA). Color represents ncRNA biotype. **G**, Distributions of gene GC content and gene length for all genes expressed in either of the two technologies. Two-sided t-test, *** $p < 0.001$. **H**, Distributions of gene length for differentially expressed genes per gene biotype between technologies. Two-sided t-test, * $p < 0.05$, *** $p < 0.001$.

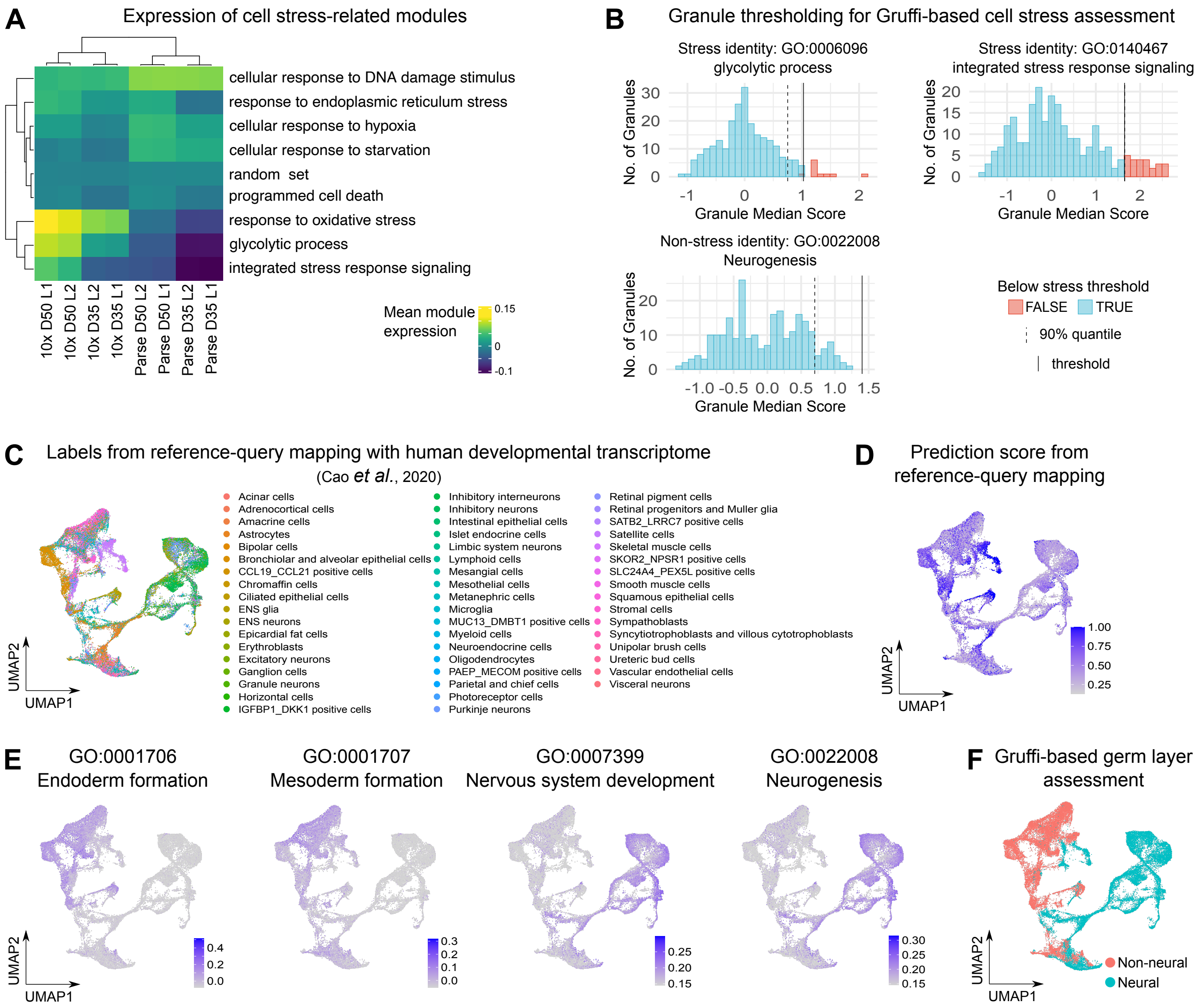
A
Expression of cell stress-related modules
cellular response to DNA damage stimulus
response to endoplasmic reticulum stress
cellular response to hypoxia
cellular response to starvation
random set
programmed cell death
response to oxidative stress
glycolytic process
integrated stress response signaling
10x D50 L1
10x D50 L2
10x D35 L2
10x D35 L1
Parse D50 L2
Parse D50 L1
Parse D35 L2
Parse D35 L1
Mean module expression
0.15
0
-0.1
B
Granule thresholding for Gruffi-based cell stress assessment
Stress identity: GO:0006096 glycolytic process
Stress identity: GO:0140467 integrated stress response signaling
Non-stress identity: GO:0022008 Neurogenesis
No. of Granules
Granule Median Score
Below stress threshold
FALSE
TRUE
90% quantile
threshold
C
Labels from reference-query mapping with human developmental transcriptome (Cao et al., 2020)
Acinar cells
Adrenocortical cells
Amacrine cells
Astrocytes
Bipolar cells
Bronchiolar and alveolar epithelial cells
CCL19_CCL21 positive cells
Chromaffin cells
Ciliated epithelial cells
ENS glia
ENS neurons
Epicardial fat cells
Erythroblasts
Excitatory neurons
Ganglion cells
Granule neurons
Horizontal cells
IGFBP1_DKK1 positive cells
Inhibitory interneurons
Inhibitory neurons
Intestinal epithelial cells
Islet endocrine cells
Limbic system neurons
Lymphoid cells
Mesangial cells
Mesothelial cells
Metanephric cells
Microglia
MUC13_DMBT1 positive cells
Myeloid cells
Neuroendocrine cells
Oligodendrocytes
PAEP_MECOM positive cells
Parietal and chief cells
Photoreceptor cells
Purkinje neurons
Retinal pigment cells
Retinal progenitors and Muller glia
SATB2_LRRC7 positive cells
Satellite cells
Skeletal muscle cells
SKOR2_NPSR1 positive cells
SLC24A4_PEX5L positive cells
Smooth muscle cells
Squamous epithelial cells
Stromal cells
Sympathoblasts
Syncytiotrophoblasts and villous cytotrophoblasts
Unipolar brush cells
Ureteric bud cells
Vascular endothelial cells
Visceral neurons
UMAP1
UMAP2
D
Prediction score from reference-query mapping
1.00
0.75
0.50
0.25
E
GO:0001706 Endoderm formation
GO:0001707 Mesoderm formation
GO:0007399 Nervous system development
GO:0022008 Neurogenesis
F
Gruffi-based germ layer assessment
Non-neural
Neural

Fig. S4. Complementary to Fig. 2. Assessment of neural lineage identity. **A**, Heatmap representing mean module expression scores of gene ontology terms related to aspects of cell stress. **B**, Histograms representing distribution of granule scores for expression of cell stress modules. Color represents stress classification. Solid black line represents stress threshold. Dashed black line represents 90% quantile of the distribution of granule expression score. Dashed blue and red lines represent median values of non- and stressed cells. **C**, UMAP plot representing cell type identity as assigned based on reference-query integration with human developmental transcriptome[29]. **D**, Feature plot showing prediction score based on reference-query integration with human developmental transcriptome. **E**, Feature plots showing module expression scores for GO terms guiding Gruffi-based lineage identity assessment. **F**, UMAP plot representing neural lineage status of cells based on Gruffi-based lineage identity assessment.

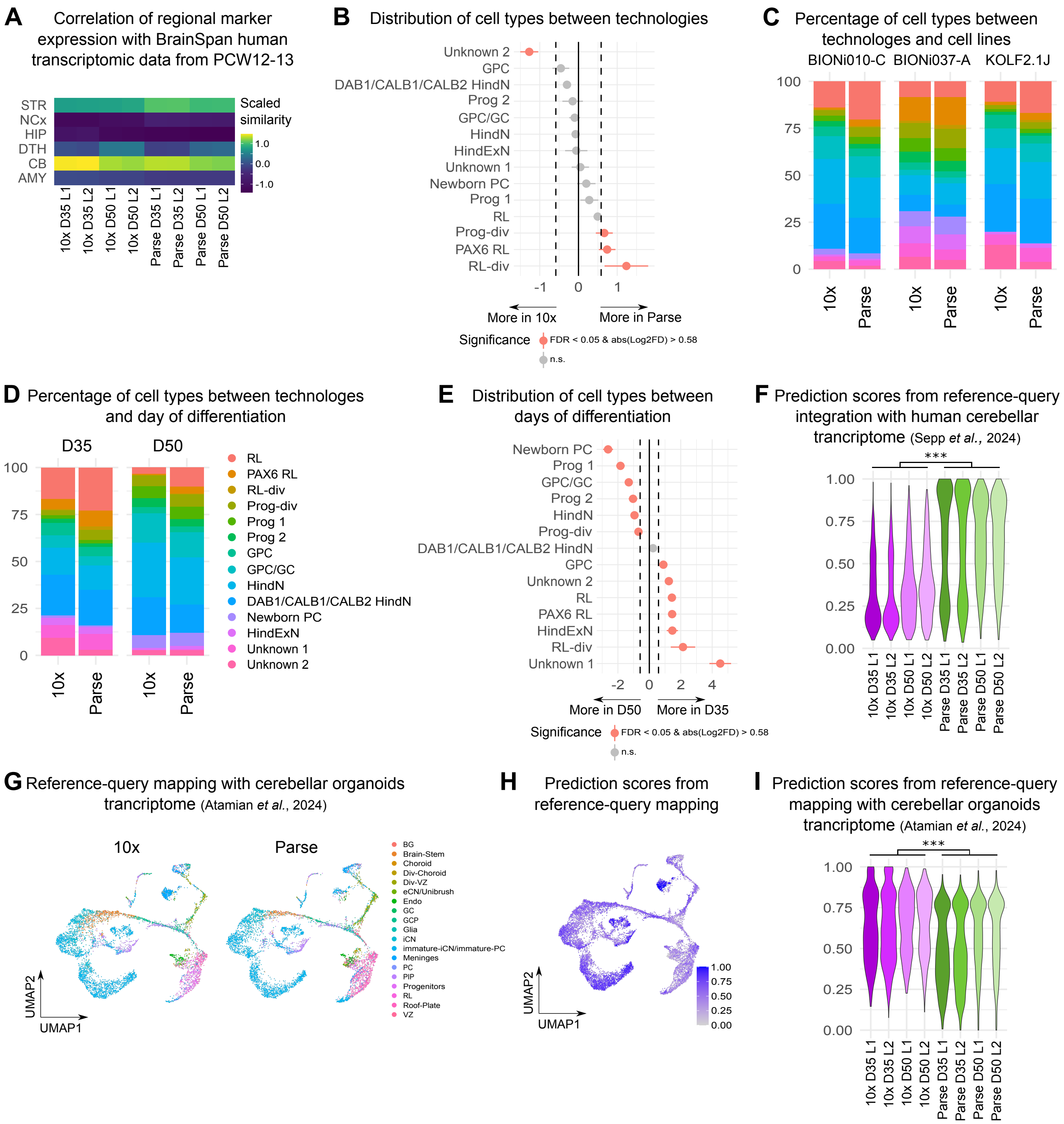
A
Correlation of regional marker expression with BrainSpan human transcriptomic data from PCW12-13
STR
NCx
HIP
DTH
CB
AMY
Scaled similarity
1.0
0.0
-1.0
10x D35 L1
10x D35 L2
10x D50 L1
10x D50 L2
Parse D35 L1
Parse D35 L2
Parse D50 L1
Parse D50 L2
B
Distribution of cell types between technologies
Unknown 2
GPC
DAB1/CALB1/CALB2 HindN
Prog 2
GPC/GC
HindN
HindExN
Unknown 1
Newborn PC
Prog 1
RL
Prog-div
PAX6 RL
RL-div
-1
0
1
More in 10x
More in Parse
Significance
FDR < 0.05 & abs(Log2FD) > 0.58
n.s.
C
Percentage of cell types between technologies and cell lines
BIONi010-C
BIONi037-A
KOLF2.1J
100
75
50
25
0
10x
Parse
D
Percentage of cell types between technologes and day of differentiation
D35
D50
100
75
50
25
0
10x
Parse
RL
PAX6 RL
RL-div
Prog-div
Prog 1
Prog 2
GPC
GPC/GC
HindN
DAB1/CALB1/CALB2 HindN
Newborn PC
HindExN
Unknown 1
Unknown 2
E
Distribution of cell types between days of differentiation
Newborn PC
Prog 1
GPC/GC
Prog 2
HindN
Prog-div
DAB1/CALB1/CALB2 HindN
GPC
Unknown 2
RL
PAX6 RL
HindExN
RL-div
Unknown 1
-2
0
2
4
More in D50
More in D35
Significance
FDR < 0.05 & abs(Log2FD) > 0.58
n.s.
F
Prediction scores from reference-query integration with human cerebellar trancriptome (Sepp et al., 2024)
***
1.00
0.75
0.50
0.25
0.00
10x D35 L1
10x D35 L2
10x D50 L1
10x D50 L2
Parse D35 L1
Parse D35 L2
Parse D50 L1
Parse D50 L2
G
Reference-query mapping with cerebellar organoids trancriptome (Atamian et al., 2024)
10x
Parse
UMAP2
UMAP1
BG
Brain-Stem
Choroid
Div-Choroid
Div-VZ
eCN/Unibrush
Endo
GC
GCP
Glia
iCN
immature-iCN/immature-PC
Meninges
PC
PIP
Progenitors
RL
Roof-Plate
VZ
H
Prediction scores from reference-query mapping
1.00
0.75
0.50
0.25
0.00
UMAP2
UMAP1
I
Prediction scores from reference-query mapping with cerebellar organoids trancriptome (Atamian et al., 2024)
***
1.00
0.75
0.50
0.25
0.00
10x D35 L1
10x D35 L2
10x D50 L1
10x D50 L2
Parse D35 L1
Parse D35 L2
Parse D50 L1
Parse D50 L2

Fig. S5. Complementary to Fig. 3. Assessment of regional identity and cell type annotation. **A**, Heatmap of scaled similarity metric of VoxHunt algorithm comparing samples with human neocortical RNA-seq data from BrainSpan using brain regional markers obtained from Mouse Brain Atlas at E13. **B**, Permutation test on cell type composition of cerebellar organoids between technologies. **C**, Stacked bar plot representing average proportion of individual cell types between technologies and cell lines. **D**, Stacked bar plot representing average proportion of individual cell types between technologies and day of differentiation. **E**, Permutation test on cell type composition of cerebellar organoids between days of differentiation. The legend is shared between C and D. **F**, Distribution of prediction scores based on reference-query integration with human cerebellar transcriptome[37]. **G**, UMAP plot representing cell type identity as assigned based on reference-query integration with cerebellar organoids transcriptome[21]. **H**, Feature plots showing prediction score based on reference-query integration with cerebellar organoids transcriptome[21]. **I**, Distribution of prediction scores based on reference-query integration with human cerebellar organoids. For B and E, differentially abundant cell types are represented in pink. Cell types with FDR less than 0.05 and absolute log2 fold change more than 0.58 were considered differentially abundant. For F and I, color represents sample identity with respect to technology (10x or Parse), day of differentiation (D35 or D50), and library (L1 or L2). For F and I three-way ANOVA, p-values represent differences between technologies, *** $p < 0.001$.

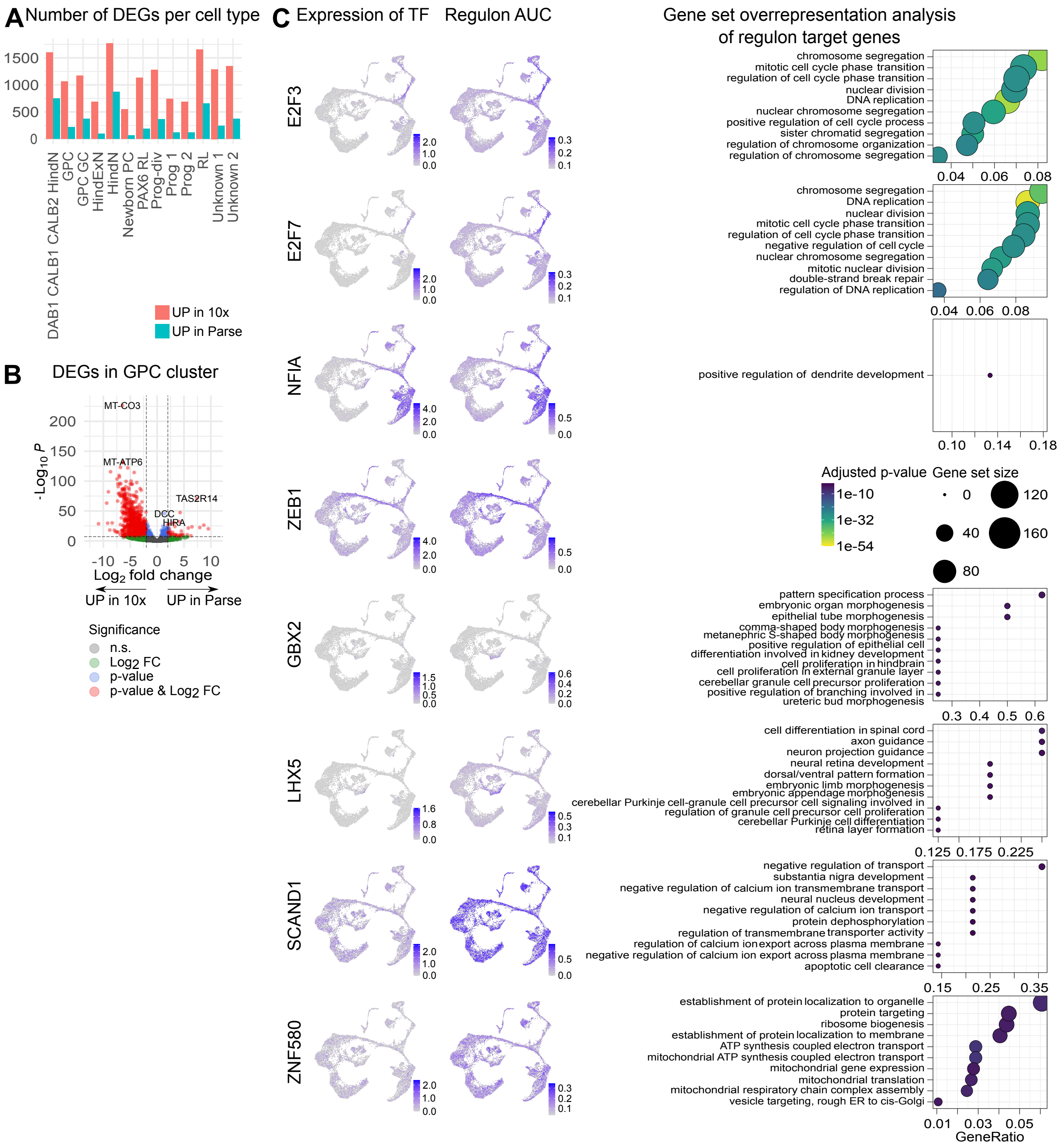
A
Number of DEGs per cell type
1500
1000
500
0
DAB1 CALB1 CALB2 HindN
GPC
GPC GC
HindExN
HindN
Newborn PC
PAX6 RL
Prog-div
Prog 1
Prog 2
RL
Unknown 1
Unknown 2
UP in 10x
UP in Parse
B
DEGs in GPC cluster
MT-CO3
MT-ATP6
TAS2R14
DCC
HIRA
200
150
100
50
0
-Log10 P
-10 -5 0 5 10
Log2 fold change
UP in 10x
UP in Parse
Significance
n.s.
Log2 FC
p-value
p-value & Log2 FC
C
Expression of TF
Regulon AUC
E2F3
E2F7
NFIA
ZEB1
GBX2
LHX5
SCAND1
ZNF580
Gene set overrepresentation analysis
of regulon target genes
chromosome segregation
mitotic cell cycle phase transition
regulation of cell cycle phase transition
nuclear division
DNA replication
nuclear chromosome segregation
positive regulation of cell cycle process
sister chromatid segregation
regulation of chromosome organization
regulation of chromosome segregation
0.04 0.06 0.08
chromosome segregation
DNA replication
nuclear division
mitotic cell cycle phase transition
regulation of cell cycle phase transition
negative regulation of cell cycle
nuclear chromosome segregation
mitotic nuclear division
double-strand break repair
regulation of DNA replication
0.04 0.06 0.08
positive regulation of dendrite development
0.10 0.14 0.18
Adjusted p-value
1e-10
1e-32
1e-54
Gene set size
0
40
80
120
160
pattern specification process
embryonic organ morphogenesis
epithelial tube morphogenesis
comma-shaped body morphogenesis
metanephric S-shaped body morphogenesis
positive regulation of epithelial cell
differentiation involved in kidney development
cell proliferation in hindbrain
cell proliferation in external granule layer
cerebellar granule cell precursor proliferation
positive regulation of branching involved in
ureteric bud morphogenesis
0.3 0.4 0.5 0.6
cell differentiation in spinal cord
axon guidance
neuron projection guidance
neural retina development
dorsal/ventral pattern formation
embryonic limb morphogenesis
embryonic appendage morphogenesis
cerebellar Purkinje cell-granule cell precursor cell signaling involved in
regulation of granule cell precursor cell proliferation
cerebellar Purkinje cell differentiation
retina layer formation
0.125 0.175 0.225
negative regulation of transport
substantia nigra development
negative regulation of calcium ion transmembrane transport
neural nucleus development
negative regulation of calcium ion transport
protein dephosphorylation
regulation of transmembrane transporter activity
regulation of calcium ion export across plasma membrane
negative regulation of calcium ion export across plasma membrane
apoptotic cell clearance
0.15 0.25 0.35
establishment of protein localization to organelle
protein targeting
ribosome biogenesis
establishment of protein localization to membrane
ATP synthesis coupled electron transport
mitochondrial ATP synthesis coupled electron transport
mitochondrial gene expression
mitochondrial translation
mitochondrial respiratory chain complex assembly
vesicle targeting, rough ER to cis-Golgi
0.01 0.03 0.05
GeneRatio

Fig. S6. Complementary to Fig. 4. Differential gene expression between technologies. **A**, Bar plot representing number of differentially expressed genes per cell type. **B**, Volcano plot representing differential gene expression in GPC cluster without genes that are exclusively expressed in one of the technologies. **C**, Feature plots showing expression of selected TFs (left column), their regulon AUC scores (middle column), and results of gene set overrepresentation analysis in TF target genes within regulons (right column). ZEB1 did not have any significantly enriched GO terms.
